# Prevalence of Vitamin-D deficiency is related to severity of liver damage in Hepatitis-C patients

**DOI:** 10.12669/pjms.36.3.1490

**Published:** 2020

**Authors:** Sadia Falak, Lubna Aftab, Muhammad Saeed, Aftab Islam

**Affiliations:** 1 Dr. Sadia Falak, PhD. School of Pharmacy, The University of Faisalabad, Faisalabad-38040, Pakistan; 2 Dr. Lubna Aftab, MBBS. Department of Biochemistry, Faculty of Sciences, University Medical and Dental College, Faisalabad-38040, Pakistan; 3 Dr. Muhammad Saeed, PhD. Department of Biochemistry, Faculty of Sciences, University Medical and Dental College, Faisalabad-38040, Pakistan; 4 Dr. Aftab Islam, MBBS. Medical Practitioner, Dar-us-Shifa Clinic, Faisalabad-38040, Pakistan

**Keywords:** Compensated cirrhosis, Decompensated cirrhosis, 25 (OH) D, Hepatitis C infection, Vitamin-D stratum

## Abstract

**Objective:**

Serum Vitamin-D plays pivotal role in inflammatory and infectious diseases; among them liver infections are more distinct. This study was aimed to determine Vitamin-D status in HCV-infected patients and healthy controls in Faisalabad, Pakistan.

**Methods:**

We performed randomized cross-sectional study of 74 individuals from 20^th^ August, 2017 to 20^th^ February 2018 at The University of Faisalabad and Dar us Shifa Clinic, Faisalabad. Fifty-one patients were hepatitis C RNA-PCR positive (22 compensated cirrhotic and 29 decompensated cirrhotic patients). In addition, 23 subjects without liver disease were recruited as healthy control. HCV RNA–PCR was performed by ARTUS ® HCV QS-RGQ V1. Vitamin-D levels were measured by chemiluminescence. SPSS version 20 was used for statistical analysis.

**Results:**

The mean level of Vitamin-D was significantly lower in HCV patients in compensated and decompensated cirrhotic patients (26.85 ng/mL & 20.65 ng/mL respectively) as compared to healthy controls (30.41 ng/mL). This study showed sub optimal level of Vitamin-D in 76.5% of HCV patients. Vitamin-D insufficiency (21-29 ng/mL) as prevalent among healthy individuals (47.8%) as well as in HCV patients (39.2%) (P < 0.001). In addition, Vitamin-D levels showed inverse relationship with more severe conditions of liver disease as 55.2% of decompensated cirrhosis patients were sufferer of Vitamin-D deficiency as compared to 13.6% deficiency of Vitamin-D in compensated cirrhotic group (P <0.0001).

**Conclusion:**

Suboptimal levels of Vitamin-D (deficiency or insufficiency) are prevalent in patients having hepatitis C infection as compared to healthy controls. Deficiency of Vitamin-D was directly associated with severity of disease.

## INTRODUCTION

Hepatitis C virus (HCV) is a significant health problem, which may be life threatening due to its severe complications. HCV accounts for over 700,000 deaths each year with more than 170 million people infected.^1^ In Pakistan, HCV prevalence is high and plays a major role in liver disease burden. In fact, it is an epidemic of mega proportions-one in every 20 is infected with HCV in Pakistan. So, there is a need to take measures for prevention and treatment of this epidemic as a national priority.^2^

It is now well established that Vitamin-D deficiency is associated with inflammatory and infectious diseases; among them liver infections are most distinct. Vitamin-D serum concentrations inversely associate with the severity of liver damage and the advancement of liver fibrosis or cirrhosis. There are various reports which show a significant association of 25-hydroxyVitamin-D levels in the serum and hepatitis C.^3^

Up to 90% of Vitamin-D (in form of cholecalciferol - D_3_) is retrieved from ultra violet B (UVB) irradiation induction in skin. Remainder is derived orally from diet or supplements.^4^,^5^ Fish, eggs and milk in the diet are considered good source of D_3_ from diet_._ Vitamin-D_2_ (ergocalciferol) is not produced in humans, and is derived from plants. Both Vitamin-D_3_ and D_2_ are physiologically inactive. The activation of Vitamin-D occurs in two steps hydroxylation reaction; first hydroxylation at 25^th^ carbon atom occurs in liver, and this is one of the reasons why Vitamin-D deficiency is related to chronic liver disease. Next hydroxylation occurs at 1^st^ carbon of calcidiol in proximal convoluted tubules of kidneys. This leads to the formation of fully activated form of Vitamin-D as 1, 25(OH) _2_ D_3_, also known as calcitriol.^6^

The estimation of Vitamin-D level is done by measuring serum concentration of both forms of 25-(OH)D_3_ and D_2,_ as assessment of serum calcitriol (activated form) is intricate due to shorter life span and low concentration. The Endocrine Society Clinical Practice Guidelines on Vitamin-D defines deficiency as <20 ng/mL, insufficiency as 21–29 ng/mL and sufficiency as >30 ng/mL of serum 25-OH-D.^4^

The prevalence of HCV infections in Faisalabad region approximately ranges up to 22% which is alarming as compared to other regions of country.^7^ So preventive measure must be taken to eradicate this epidemic. As Vitamin-D deficiency is strongly associated to HCV infection, hence this present project was designed to get insight at the status of Vitamin-D in HCV patients, investigating the prevalence of 25 (OH) D_3_ with different cohorts of HCV patients.

## METHODS

To know the association of Vitamin-D status with Hepatitis C virus, seventy-four (n=74) subjects (using empirical approach) were enrolled from 20^th^ August, 2017 to 20^th^ February 2018.^8^ This randomized cross-sectional study was conducted at The University of Faisalabad and Dar us Shifa clinic, Faisalabad. Due ethical clearance was taken from Research and Ethical Review Committee, The University of Faisalabad, Pakistan (Ref. No. TUF/Dean/2017/53 dated July 12, 2017). Informed consent was taken from all the participants. The subjects were assigned to two clinical groups, compensated cirrhotic patients (n=22) with hepatitis C RNA-PCR positive but not having ascites and decompensated cirrhotic group (n=29) with hepatitis C RNA-PCR positive, ascites and/or other clinical signs of decompensated cirrhosis like jaundice, hematemesis etc. In addition, 23 control subjects were also employed in this study who were seronegative for anti-HCV antibody. All participants had no other known chronic illness and no Vitamin-D supplementation for last six months.

### HCV RNA amplification and detection

HCV RNA was extracted by using “QIAsymphony SP” automation work station by QIAGEN GmbH. It works on the principle of magnetic particle-based automated RNA extraction. RNA was amplified and detected using artus HCV RG RT-PCR kit constituted a ready-to-use system for the detection of HCV RNA using polymerase chain reaction (PCR) on Rotor-Gene Q Plex - MDx, a Real-time PCR cycler.

### 25-OH-Vitamin-D measurement

25-OH-Vitamin-D serum concentrations were evaluated from patients and control by enhanced chemiluminescence method, using Vitros ECi immunodiagnostic system (Johnson & Johnson).^9^ Sample, dissociation buffer and conjugate buffer were sequentially added to Vitros® microwell coated with a monoclonal antibody (MAb). Dissociated Vitamin-D competed with the conjugate Vitamin-D to bind with the antibody. After an incubation period unbound materials were washed away. An enhaced chemiluminescence substrate was then added and light emission measured. The data was expressed in ng/mL.

### Statistical Analysis

All statistical analyses were done using SPSS Version 20.0 (SPSS, Chicago, IL). Categorical variables were presented in frequency and percentage while quantitative variables were presented by mean±SD. To compare the difference between group’s χ2 tests and Kruskal- Wallis tests were applied for categorical data. Continuous variables were analyzed using Student’s *t* test or Mann-Whitney test with normal or skewed distribution. Figures were drawn using GraphPad Prism 8 (GraphPad Software Inc., California, USA).

## RESULTS

A total of 74 participants were recruited in this study accorging to empirical approach. Based on clinical investigations, and anti-HCV titers, subjects were classified into control healthy subjects (n=23), and hepatitis C patients (n=51). HCV group was categorized in two subgroups compensated cirrhosis (cHCV) (n=22) and decompensated cirrhosis (dHCV) (n=29) as shown in Table-I. All the subjects were aged between 20-65 years. Healthy controls were younger than HCV patients (P<0.001). Mean age of the controls was 36.09±9.125years, HCV patients with compensated cirrhosis 48.68±12.442, and decompensated cirrhosis 50.69± 8.410. Majority of both controls and patients were females (60.2% and 60.8%respectively). However, the difference was non-significant on the basis of gender (p=0.729). No significant differences were observed among different study groups for joint pain (p=0.166) as normal population also suffers from Vitamin-D insufficiency. For the biochemical parameters, there was a significant change in jaundice, hematemesis and blood transfusion activity (P < 0.068) (Table-I).

**Table-I:** Demographic and clinical parameter of HCV and control subjects.

Characteristics	Control (n=23)	Compensated Cirrhosis (n= 22)	Decompensated Cirrhosis (n =29)	P-value
Age (Mean, range)	36.09(20-50)	48.68(29-65)	50.69 (35-65)	< 0.001
Gender (Male/Female)	9/14	10/12	10/19	0.729
Joint Pains (No/Yes)	6/17	2/20	9/20	0.166
Ascites (No/Yes)	22/1	22/0	29/0	< 0.001
Jaundice (No/Yes)	23/0	21/1	6/23	< 0.001
Hematemsis (No/Yes)	23/0	20/2	12/17	< 0.001
Blood Transfusion (No/Yes)	19/4	14/8	15/14	0.068
HCV RNA PCR QL (No/Yes)	23/0	0/22	0/29	< 0.001
Serum 25(OH)D ng/mL	30.4130	26.8500	20.6483	< 0.001

P-values were calculated by Chi-square test and Kruskal-Wallis test where appropriate.

**Fig.1:**
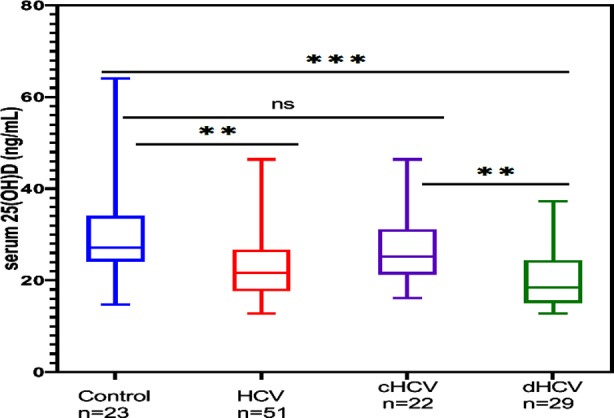
Vitamin-D levels in healthy subjects, HCV patients and subgroups. HCV: Hepatitis C positive, cHCV: compensated cirrhosis, dHCV: decompensated cirrhosis. P-values were calculated by Mann-Whitney Wilcoxon test.

The 25 (OH) Vitamin-D was significantly lower in HCV patients as compared to healthy subjects (Table-I; Fig.1). In the HCV patients’ subgroups, Vitamin-D level in control and cHCV patients had no significant difference (P= 0.117) as compared to control vs dHCV (P < 0.001)

**Fig 2:**
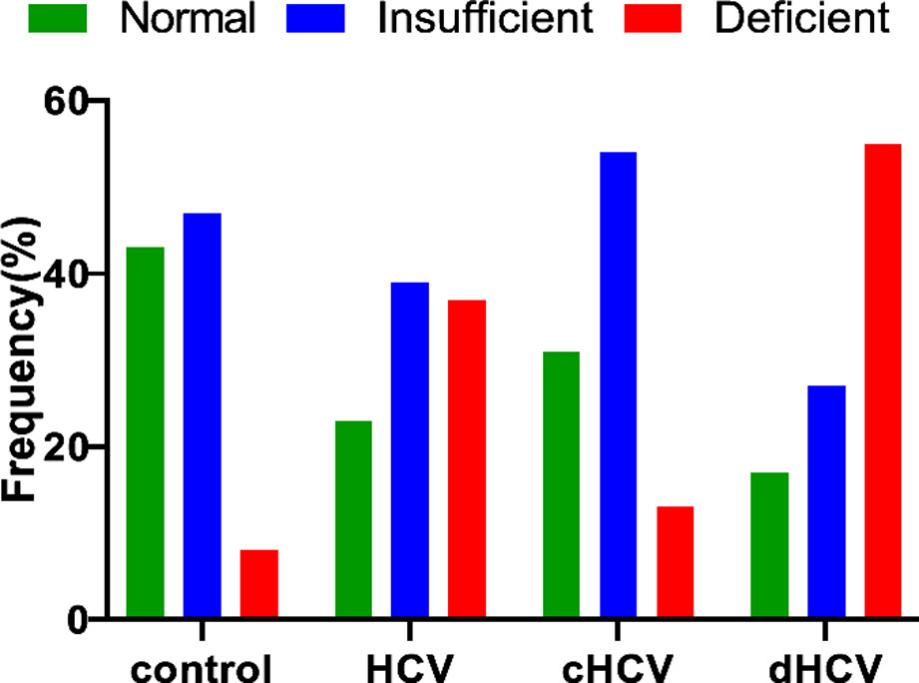
Frequency distribution of Vitamin-D levels in healthy subjects. HCV patients and subgroups; HCV: Hepatitis C positive, cHCV: compensated cirrhosis, dHCV: decompensated cirrhosis.

When Vitamin-D distribution was classified according to the Endocrine Society Clinical Practice Guidelines, majority of the study population were found to be suffering from insufficient level of Vitamin-D. On average, healthy control group barely falls in sufficient level of Vitamin-D category, while HCV patients with compensated cirrhosis group had insufficient level of 25 (OH) Vitamin-D and decompensated group were low in deficient category of 25 OH) Vitamin-D. Vitamin-D deficiency and insufficiency were significantly associated with healthy control and all HCV patients and also in subgroups (P<0.0001 for all groups and P=0.03 control vs. HCV patients) (Table-II; Fig.2).

**Table-II:** Frequency distribution of HCV-infected patients and healthy controls with Vitamin-D stratum.

Vitamin-D stratum	Control (n=23) n (%)	HCV (n= 51) n (%)	Compensated Cirrhosis (n= 29) n (%)	Decompensated Cirrhosis (n =22) n (%)
Deficient	2 (8.7%)	19 (37.3%)	3 (13.6%)	16(55.2%)
Insufficient	11(47.8%)	20 (39.2%)	12 (54.5%)	8(27.6%)
Normal	10 (43.5%)	12 (23.5%)	7(31.8%)	5(17.2%)

P = <0.0001 (Kruskal-Wallis test for all groups including control, compensated and decompensated cirrhosis). P = 0.031 (Chi-square test: Control vs HCV patients).

Out of 51 patients only 23.5% of the population had normal level of Vitamin-D, 37.3% patients had deficiency while 39.2% had insufficiency of 25 (OH) D. In subgroups more significantly deficient level of 25 (OH) D was observed in decompensated cirrhosis HCV patients (55.2%) as compared to compensated Cirrhosis patients (13.6%).

## DISCUSSION

Hepatitis C virus infection is one of the significant public health issues which play a major role in liver disease burden globally. The liver cirrhosis being 4^th^ foremost cause of the death in many geographical regions of the world is majorly attributed by HCV infection.^2^ It has been reported that chronic liver disease sufferers are most likely to develop deficiency of Vitamin-D. Role of Vitamin-D is not only restricted to its classical action in bone homeostasis and calcium regulation; this vitamin is also involved in a plethora of physiopathology of various diseases as an immune modulator in both innate and adaptive immune mechanism.^10^,^11^ Approximately 93% prevalence of Vitamin-D deficiency has been reported in chronic liver diseases, while 1/3 of this accounts to be severe deficit.^4^

Therefore, the present study was planned to observe the status of 25 (OH) D in HCV patients comprising of two groups, compensated liver cirrhosis and decompensated liver cirrhosis according to progression of liver disease. This study shows 76.5% of sub optimal levels of Vitamin-D among HCV patients (37.3% deficit and 39.2% insufficient level respectively) as compared to healthy individuals. These results strongly corroborate the findings of published data for Vitamin-D deficiency in liver disease patients. Kumar et al., found suboptimal level of Vitamin-D in 80% cirrhotic patients.^12^ One more study conducted in Rawalpindi, Pakistan by Hamid & colleagues found that 78% of the 120 cirrhotic patients of HCV suffer from inadequate level of Vitamin-D.^13^ However contradictory to these findings previously reports showed 90% patients suffer from low level of Vitamin-D (31% deficit in and 59% insufficient).^14^ This was because severity and prognosis of HCV leads to dysfunctioning of endogenous Vitamin-D synthesis as a result of reduced synthesis of 7-dehydrocholestrol. One more contributory factor for low serum Vitamin-D may be limited exposure of patients to sunlight.^15^

Vitamin-D levels showed inverse relationship with more severe conditions of liver disease as 55.2% of decompensated cirrhosis patients were sufferer of Vitamin-D deficiency compared to 13.6% deficiency of Vitamin-D in compensated cirrhotic group (shown in Fig.2). These findings suggest that serum Vitamin-D levels contribute significantly to the clinical courses of HCV infection. As pluripotent in nature, Vitamin-D is involved in immune modulator and anti-inflammation activities. So it reduces HCV core antigen and virus particle assembly. Vitamin-D deficiency may also lead towards increased viral load, increased inflammation and damage to liver.^16^ However Vidot and colleagues reported that Vitamin-D insufficiency was not associated with chronic liver diseases and complications.^17^ Similarly Basile and his team didn’t find any substantial differences in serum 25 (OH) D3 and Vitamin-D binding protein (DBP) levels between HCV and healthy controls.^18^ On the other hand role of Vitamin-D supplementation is still unclear, as inconsistent results have been observed in assessing the relationship of Vitamin-D supplementation along other therapies on sustained virologic response (SVR).^19^,^20^

We observed 39.2% insufficiency of 25 (OH) D in HCV patients. Ladero et al. also noted 40% of insufficient levels in their study population.^21^ Mandorfer & colleagues found 57% insufficiency of serum Vitamin-D.^22^ These collective studies correspond with our results that Vitamin-D is insufficient in patients suffering from Hepatitis C virus infection. However insufficiency of Vitamin-D was also observed in normal healthy subjects. A larger scale study was conducted by Riaz and his team to determine the prevalence of Vitamin-D deficiency in Pakistan which shows majority of the population has Vitamin-D deficiency.^23^ This is the reason that joints pain was reported by the healthy individuals as well as HCV infected patients. A number of factors can be responsible for heterogeneity in suboptimal Vitamin-D levels including racial differences, BMI and dietary factors.^24^

### Limitation of this study

It includes small sample size and mono center study that restricts the generalizability of the results. Also this study was conducted in short time period (specific season). Further prospective studies are needed to better define the role of Vitamin-D in HCV patients based on large scale, multicenter and long duration.

## CONCLUSION

We observed low level of Vitamin-D concentration in HCV patients as compared to healthy controls; 76.5% HCV patients had sub optimal level of Vitamin-D. The severity of deficiency of Vitamin-D was directly associated with severity of disease. The percentage of patients having deficiency of Vitamin-D was more in decompensated group as compared to compensated cirrhotic group. Insufficiency of 25 (OH) D was also observed in healthy control population along with HCV positive patients. So it is recommended that estimation of Vitamin-D should also be done along with screening test for HCV. Vitamin-D supplements and lifestyle modifications can improve the Vitamin-D status in general and delay the progression of liver disease in HCV patients. That being discussed, further, large scale, multicenter and long duration studies are still needed for affirmation of these findings.

### Author`s Contribution:

**SF** main Author, Manuscript writing, review of literature, data analysis, discussion.

**LA** did study designing, data collection& editing of manuscript.

**MS** conceived the idea of manuscript and was in charge of overall direction and planning.

**AI** provided guidelines and helped in data collection.

**SF** takes the responsibility and is accountable for all aspects of the work in ensuring that questions related to the accuracy or integrity of any part of the work are appropriately investigated and resolved.
